# A rare case report of successful surgical treatment of diaphragmatic pregnancy after chemotherapy

**DOI:** 10.3389/frph.2025.1631911

**Published:** 2025-10-16

**Authors:** Bin Li, Aihong Duan, Dandan Guo, Ruifeng Qin, Tiantian He

**Affiliations:** ^1^ Department of Gynecology, Handan Central Hospital, Handan, China; ^2^ Department of General Surgery, Handan Central Hospital, Handan, China

**Keywords:** diaphragmatic pregnancy, chemotherapy, surgical treatment, methotrexate, ectopic pregnancy

## Abstract

**Background:**

Diaphragmatic pregnancy is a rare type of ectopic pregnancy, and its clinical manifestations are complicated and easy to misdiagnose, which presents great challenges for clinical diagnosis and treatment. We report a case of diaphragmatic pregnancy and describe its difficult but successful diagnosis and treatment in detail.

**Patient presentation:**

A 34-year-old woman from northern China was admitted to the hospital with delayed menstruation and right upper abdominal pain with right shoulder pain (Kehr's sign). An initial emergency laparoscopy for suspected ruptured ectopic pregnancy revealed hemoperitoneum but failed to identify the ectopic pregnancy tissue, likely due to an incomplete surgical survey. Subsequent serial *β*-hCG monitoring showed a persistent rise, and MRI localized the ectopic pregnancy tissue at the outer edge of the right lobe of the liver. Conservative treatment with methotrexate and 5-fluorouracil chemotherapy was given in time, which induced a significant biochemical response. However, due to the worsening of abdominal pain despite declining *β*-hCG levels, indicating a persistent risk of rupture, definitive laparoscopic resection was performed. Intraoperatively, the pregnancy tissue was found to be implanted on the surface of the diaphragm, and the pregnancy tissue was successfully removed completely from the diaphragm with little intraoperative bleeding. Postoperative pathology results confirmed the presence of pregnancy chorionic tissue. The patient recovered well without complications and was discharged 5 days after surgery.

**Conclusion:**

This case highlights that Kehr's sign is a crucial diagnostic clue for upper abdominal ectopic pregnancy. A systematic survey of the entire abdomen, including the diaphragm, is mandatory during laparoscopy to avoid diagnostic omission. The sequential combination of chemotherapy and surgical treatment may represent an effective strategy to minimize surgical risk and optimize outcomes for this high-risk condition.

## Introduction

Ectopic pregnancy refers to the fertilization of eggs in places other than the uterine cavity, the most common of which is tubal pregnancy (1). Abdominal pregnancy is rare, accounting for approximately 1.4% of ectopic pregnancies (2), whereas diaphragmatic pregnancy is a special case of abdominal pregnancy. Diaphragmatic pregnancy is often misdiagnosed and underdiagnosed due to its special location and complex clinical manifestations.

A search of the PUBMED literature from 1976 to April 2025 using the search terms “nontubal pregnancy” or “abdominal pregnancy” or “diaphragmatic pregnancy” identified a number of published reports, while documented cases of ectopic pregnancy on the diaphragmatic surface are uncommon (3–8). In this article, we report a case of diaphragmatic pregnancy that was difficult but successfully diagnosed and treated, aiming to improve clinicians' understanding of diaphragmatic pregnancy and explore its effective diagnosis and treatment strategies, and may provide guidance for clinical practice.

## Case presentation

A 34-year-old woman (gravida 3, para 3, abortus 4) from northern China with a history of three previous cesarean sections and one unilateral salpingectomy for ectopic pregnancy (details unknown), was admitted to our department at 21:00 on 30 March 2025. The patient complained of delayed menstruation for 40 days and right upper abdominal pain with right shoulder discomfort (Kehr's sign) for three days, which was aggravated for two hours, and there was no history of abnormal vaginal bleeding. In the emergency department, the patient was examined for *β*-hCG (4,445 mIU/ml) and progesterone (20.39 ng/mL), the blood count showed hemoglobin at 89 g/L. Upper abdominal ultrasound revealed perihepatic fluid, lower abdominal ultrasound revealed pelvic fluid, an abnormal echogenic mass in the left adnexal region, and no gestational echogenicity in the uterine cavity. On admission, her vital signs were stable: blood pressure 125/80 mmHg, heart rate 88 beats per minute, respiratory rate 16 breaths per minute, and oxygen saturation 99% on room air.

Immediately after admission to our department, a puncture of the posterior vaginal dome was performed, and noncoagulated blood was punctured out. Considering the rupture of the ectopic pregnancy with intra-abdominal hemorrhage, laparoscopic exploratory surgery was performed at night as an emergency. During the operation, the amount of accumulated pelvic-abdominal blood was approximately 1,000 ml, with the absence of left ovarian and fallopian tube, and slight thickening of the right fallopian tube and suspicious for being the site of ectopic implantation, and a luteal cyst with a size of approximately 3 cm*3 cm*3 cm was observed in the right ovary, and hemorrhage was also noted in the right upper abdomen, below the diaphragm. A meticulous examination of the pelvic cavity and upper abdomen was performed. Although the subdiaphragmatic hematoma was carefully aspirated during the operation, they were unable to conduct a thorough exploration of the upper abdomen and subdiaphragmatic regions. As a result, no ectopic pregnancy tissue was identified, and the origin of the intra-abdominal hemorrhage remained undetermined at that time. The patient's family was informed of the intraoperative situation and agreed to perform a right salpingectomy, with one postoperative abdominal drain in place.

**Figure 1:**
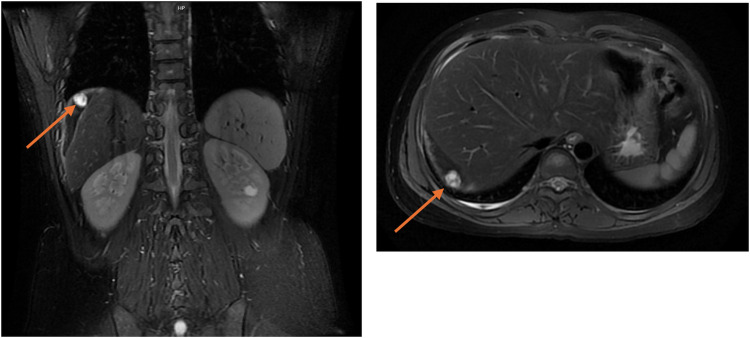
Abdominal MRI showed ectopic pregnancy tissue combined with hemorrhage visible in the periphery of the right lobe of the liver, with a maximum diameter of approximately 1.6 cm, as indicated by the arrow.

After the operation, the patient's right upper abdominal pain (Kehr's sign) alleviated slightly. On the second postoperative day, the patient's *β*-hCG level (5,400 mIU/mL) was higher than preoperative levels, indicating persistent ectopic pregnancy. Both the patient and their family were informed of the condition and expressed understanding. Conservative treatment involving an intramuscular injection of 80 mg of methotrexate (MTX) was administered. On the 4th postoperative day, the blood *β*-hCG level was 5,933 mIU/mL, and on the 5th postoperative day, the blood *β*-hCG level was 6,858 mIU/mL. This persistent rise suggested an inadequate response to MTX. The patient and their family were informed of this, and further examinations were arranged, including a pulmonary CT scan and a contrast-enhanced abdominal MRI scan. The lung CT scan revealed no abnormalities, eliminating the possibility of gestational trophoblastic disease. However, the abdominal enhanced MRI revealed that the size of the ectopic pregnancy tissue was approximately 1.6 cm at the outer edge of the right lobe of the liver, accompanied by peripheral clots, which was considered to be the tissue of the abdominal cavity pregnancy (Figure 1). The patient was in a stable condition, with no bloody drainage from the abdominal catheter, and slight relief of pain in the right upper abdominal pain. Following consultation with the patient and their family regarding further conservative or surgical treatment, they opted for conservative management initially. As MTX-based conservative treatment yielded suboptimal results, therapy was switched to intravenous mono-chemotherapy with 5-fluorouracil (5-FU) at a dose of 1.25 mg per day for 6 days. The blood *β*-hCG levels were rechecked every other day and decreased to 5,310 mIU/mL, 4,231 mIU/mL, and 1,886 mIU/mL, indicating a significant downward trend.

**Figure 2:**
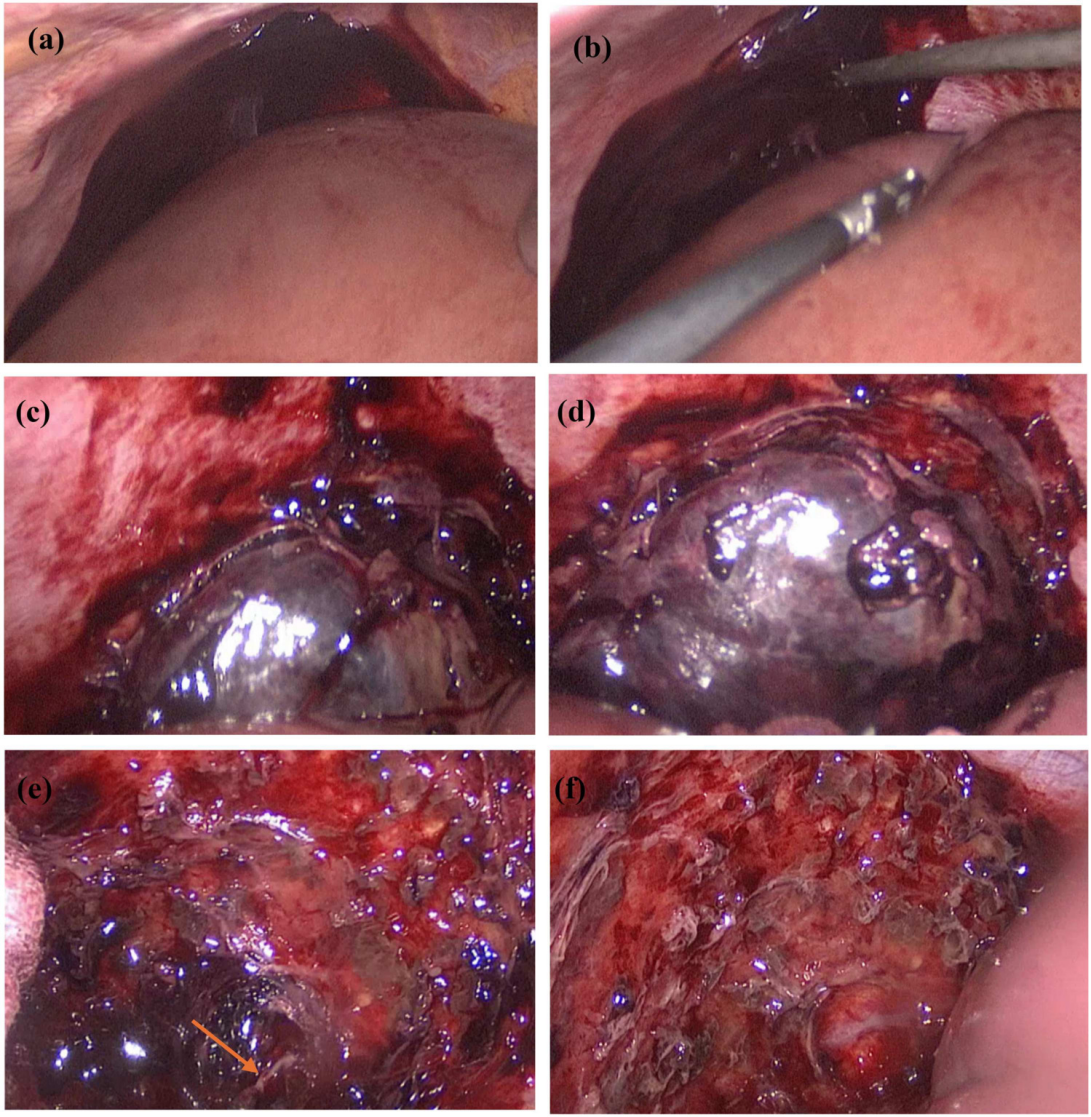
Diaphragmatic pregnancy undergoing laparoscopic surgery. **(a)** shows the clot in the epigastric region seen on initial entry into the abdominal cavity; **(b)** intraoperative suction of the clot; **(c)** exposure of the pregnancy tissue after suction of the clot; **(d)** separation of the clot from the adhesion to the liver surface to fully expose the pregnancy tissue; **(e)** vascularity on the surface of the diaphragm visible after removal of the pregnancy tissue as shown by the arrows in the figure; **(f)** complete removal of the pregnancy tissue.

On the 7th day of conservative treatment, the patient experienced worsening right upper abdominal pain with shoulder discomfort (Kehr's sign). Following a multidisciplinary consultation and discussion involving general surgery, imaging, and gynecology, laparoscopic surgery was recommended to remove the abdominal pregnancy tissue. The patient and their family also requested surgical intervention. A second laparoscopic surgery was performed on 11 April 2025. During the operation, the pregnancy tissue and the clot were located between the top of the right lobe of the liver and the diaphragm. Careful dissection revealed that the tissue was implanted on the diaphragmatic surface with a visible vascular supply (Figure 2). The pregnancy tissue and clots were completely removed, and the blood vessels on the surface of the diaphragm were coagulated using bipolar electrocautery.

**Figure 3:**
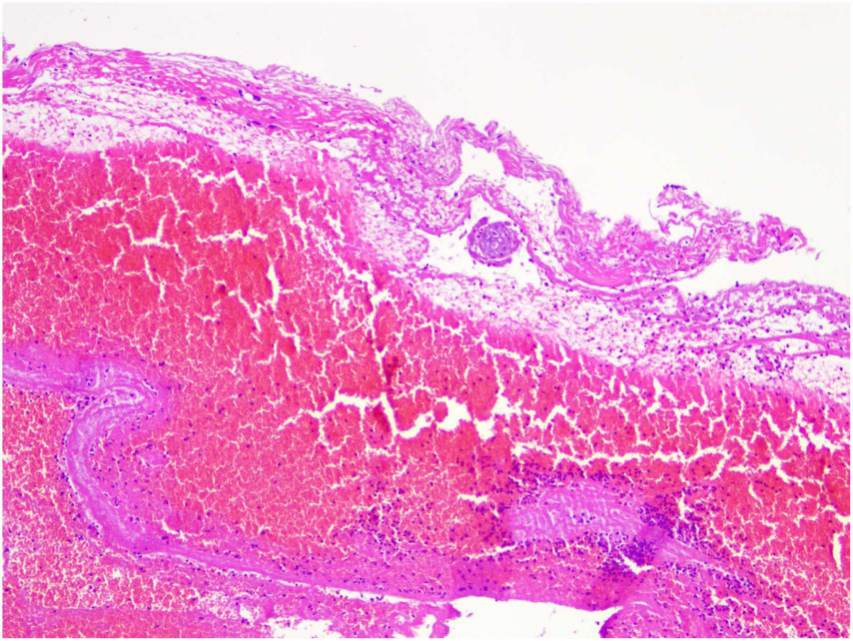
Pathological microscopy showing hemorrhagic exudate and degenerated villous tissue (HE stain, × 100).

**Figure 4:**
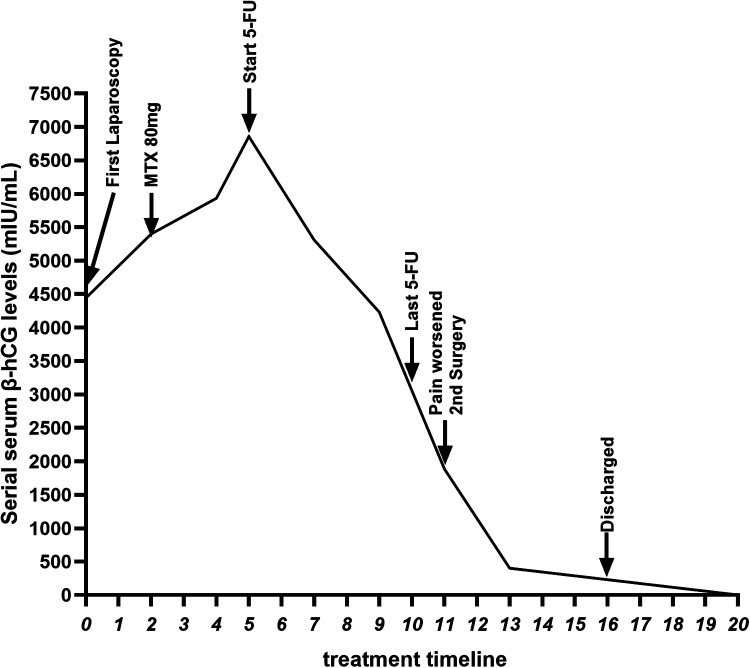
Serial serum *β*-hCG levels and treatment timeline.

Postoperatively, the patient's right upper abdominal pain (Kehr's sign) was relieved, and the patient's blood *β*-hCG value was 402.5 mIU/mL on the 2nd postoperative day. Postoperative pathology results confirmed the presence of pregnancy chorionic tissue (Figure 3). The patient tolerated chemotherapy and two surgeries well and the postoperative incision healed satisfactorily. The patient recovered well after surgery and was discharged 5 days after surgery. No adverse events or complications occurred during treatment or follow-up, the patient expressed high satisfaction with the treatment process and nursing care. The postoperative *β*-hCG level gradually decreased to within the normal range, indicating that the pregnant tissue had been completely removed (Figure 4).

## Discussion

Diaphragmatic pregnancy is a rare type of ectopic pregnancy whose pathogenesis is not fully understood. Owing to the close proximity of the diaphragm to the abdominal organs, the clinical manifestations of diaphragmatic pregnancy are often similar to those of gastrointestinal disorders, resulting in a high rate of misdiagnosis. This case highlights that recognition of specific clinical clues—particularly Kehr's sign (referred shoulder pain)—is crucial for timely diagnosis and should prompt immediate evaluation for upper abdominal pathology (9, 10). The initial diagnostic laparoscopy, though indicated for acute hemoperitoneum, failed to identify the ectopic pregnancy tissue because a systematic exploration of the upper abdomen and diaphragmatic surfaces was not performed. This omission, rather than surgical inexperience, highlights a critical procedural gap in standard laparoscopy for pregnancy of unknown location. The subsequent successful diagnosis was achieved through persistent serial *β*-hCG monitoring, advanced cross-sectional imaging (MRI), and timely multidisciplinary consultation. Consequently, this case unequivocally demonstrates that a high index of suspicion, coupled with meticulous radiographic evaluation and a mandatory systematic survey of the entire abdomen during laparoscopy, is essential to avoid diagnostic delay.

The pathogenesis of ectopic implantation on the diaphragmatic surface remains theoretical but is thought to occur through two primary mechanisms (11, 12). The most widely accepted theory is that of secondary abdominal implantation following tubal abortion or rupture (11). In this scenario, a tubal ectopic pregnancy is dislodged, and the viable trophoblastic tissue is transported by peritoneal fluid currents. These currents naturally flow towards the diaphragm via the paracolic gutters, particularly the right side, which may explain the laterality of our case. The richly vascularized diaphragmatic peritoneum then provides a suitable bed for re-implantation and proliferation. A less likely but possible mechanism is the transperitoneal migration of a fertilized ovum (12). Here, the fertilized ovum is theorized to bypass the fallopian tube entirely, migrating through the peritoneal cavity before implanting on the diaphragm, though this is considered far less common.

In reviewing the literature on diaphragmatic pregnancy, it is first noteworthy that the diagnostic dilemma posed by diaphragmatic pregnancy is consistently highlighted in the literature, often culminating in initial exploratory surgery that fails to identify the gestation (6). Similar to our case, previous reports describe patients presenting with acute abdominal pain and hemoperitoneum, leading to urgent laparoscopy where the diagnosis was missed due to the failure to systematically examine the upper abdomen (6). This recurring theme underscores that the key to preoperative suspicion lies not in routine pelvic imaging, but in recognizing atypical signs such as Kehr's sign (referred shoulder pain)—a finding underreported in prior cases but pivotal in our diagnosis—which should mandate cross-sectional imaging (CT/MRI) before any surgical intervention. Secondly, the management of diaphragmatic pregnancy has primarily involved two strategies: primary surgical resection (4, 5, 8) or, in cases of methotrexate resistance or patient refusal of surgery, ultrasound-guided microwave ablation (7). While effective, these approaches represent binary choices. Our case introduces a novel third pathway: a planned sequential protocol of neoadjuvant chemotherapy followed by delayed surgery. Unlike Qian et al. (7) who employed microwave ablation after failed MTX therapy, we utilized 5-FU mono-chemotherapy to reduce trophoblastic activity and vascularity, creating safer surgical conditions. This approach differs fundamentally from the primary resection performed by Dennert et al. (4) and Chen et al. (5), as it intentionally delays surgery to optimize conditions and minimize the risk of catastrophic hemorrhage, a significant concern in these highly vascular lesions. Finally, while existing literature establishes a framework for treating diaphragmatic pregnancy (3–8), our experience demonstrates that a multimodal, sequential strategy may be superior to a single-modality approach. By integrating the biochemical response to chemotherapy with the definitive benefits of surgery, we propose a new paradigm that maximizes patient safety and optimizes surgical outcomes for this high-risk condition.

Chemotherapeutic agents, such as MTX and 5-FU, have been widely used in the conservative management of ectopic pregnancy by inhibiting the proliferation of trophoblast cells and inducing their apoptosis (13–15). In this case, we employed this approach, utilizing 5-FU which achieved its intended biochemical effect as evidenced by a significant decline in *β*-hCG levels. However, the decision to proceed with surgical intervention was ultimately dictated by the patient's adverse clinical trajectory. Despite the favorable biochemical response, she experienced a worsening of right upper quadrant and shoulder pain. This symptom progression, indicative of potential impending rupture or hemorrhage, presented an unacceptable and escalating risk that necessitated definitive management. Furthermore, awaiting the mass's complete resolution through conservative means would have prolonged this period of vulnerability. The patient's preference and involvement in decision-making were important factors in determining the treatment approach. Consequently, surgery was elected not only to eliminate the immediate source of symptoms and prevent a life-threatening complication but also to provide histopathological confirmation. This case underscores that for complex abdominal ectopic pregnancies like those on the diaphragm, worsening clinical symptoms must supersede reassuring biochemical markers in the clinical decision-making algorithm, and the efficacy and safety of chemotherapeutic agents as a standalone treatment for this specific condition still require further validation.

## Conclusion

In conclusion, this case highlights that Kehr's sign is a critical clue for diaphragmatic pregnancy, mandating systematic upper abdominal exploration during laparoscopy. We demonstrate a novel sequential strategy: neoadjuvant chemotherapy effectively reduced the trophoblastic activity and vascularity, enabling safe laparoscopic resection. This approach prioritizes risk mitigation over biochemical response alone, offering a new paradigm for managing high-risk ectopic pregnancies.

## Data Availability

The raw data supporting the conclusions of this article will be made available by the authors, without undue reservation.
